# Development and evaluation of new nanoformulated thiolactone derivatives for enhanced disruption of pseudomonal biofilms†

**DOI:** 10.1039/d5ra01831e

**Published:** 2025-06-24

**Authors:** Mohamed K. Gaballah, Shaimaa A. Khalid, Mohamed A. Seleem, Amr K. A. Bass, Ibrahim M. El-Sherbiny, Abdelrahman S. Mayhoub

**Affiliations:** a Center of Certified Reference Materials (CRM), Zewail City of Science and Technology 6th of October City Giza 11566 Egypt amayhoub@zewailcity.edu.eg; b Nanomedicine Laboratories, Center for Materials Science, Zewail City of Science and Technology 6th October City 12578 Giza Egypt ielsherbiny@zewailcity.edu.eg; c Reference Lab for Safety Analysis of Food of Animal Origin, Animal Health Research Institute (AHRI), Agricultural Research Center (ARC) Dokki Egypt; d Department of Pharmaceutical Organic Chemistry, Faculty of Pharmacy, Al-Azhar University Cairo 11884 Egypt; e Department of Pharmaceutical Chemistry, Faculty of Pharmacy, Minufia University Shibin-Elkom 32632 Egypt

## Abstract

*Pseudomonas aeruginosa* (*P. aeruginosa*) is a formidable, antibiotic-resistant pathogen responsible for severe infections, particularly due to its ability to form protective biofilms mediated by the quorum sensing (QS) system, a cell-to-cell communication mechanism essential for biofilm formation and virulence. Herein, we developed a novel nanoformulation of thiolactone derivatives designed to target the QS system of *P. aeruginosa*. Specifically, chlorothiolactone (CTL) compounds (*m*CTL, 4a and *p*CTL, 4b) were encapsulated within biocompatible pluronic nanoparticles to enhance their delivery and efficacy. Our nanoformulation demonstrated efficient delivery of the designated molecules, leading to inhibition of the LasR receptor, a key regulator of QS, and subsequent disruption of biofilm formation. Our results revealed that the nanoparticle-formulated CTL derivatives exhibited superior activity in influencing the kinetics of *P. aeruginosa* biofilm and suppressing the virulence factors of *P. aeruginosa*, including pyocyanin and rhamnolipid production, compared to their free counterparts. Preliminary mechanistic studies indicated that the nanoformulation significantly reduced exopolysaccharide production, a critical component for biofilm integrity. Collectively, these findings underscore the potential of 4a-NPs and 4b-NPs as promising therapeutic candidates for combating *P. aeruginosa* infections by targeting its QS-mediated biofilm formation and virulence.

## Abstract 2

Experimental representation for nanoencapsulation of thiolactone derivatives and their efficiency against *Pseudomonas aeruginosa* biofilm.

## Graphical abstract

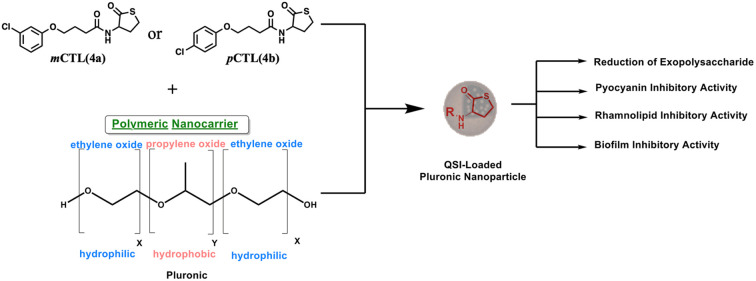

## Introduction

*Pseudomonas aeruginosa* is a serious, antibiotic-resistant Gram-negative bacterium that causes severe infections.^1^ It is also a major healthcare concern owing to its ability to resist a wide range of antibiotics, including the last resort of carbapenems and cephalosporins.^2^ This has led to its inclusion in the 2024 World Health Organization's (WHO) priority pathogen list.^3^ Life-threatening *P. aeruginosa* infections include ventilator-associated pneumonia (VAP) and bloodstream infections (BSIs),^4^ with mortality rates exceeding 40% for VAP.^5^ The mortality rate for BSIs is approximately 27%.^6^ The COVID-19 pandemic worsened the situation due to factors such as prolonged critical illness, intensive steroid use, and immune-suppressing drug use during this period.^7^

Exposure to antibiotics, starvation, or changes in pH can trigger bacterial cells to develop new, non-mutational ways to resist the pressure of external treatment. In this context, forming biofilms is the most common approach.^8^ Bacterial biofilms are slimy, protective layers that shield bacteria from antibiotics and the immune system, making infections persistent and difficult to treat.^9,10^ In this regard, biofilms play a crucial role in the pathogenesis of *P. aeruginosa*, rendering it resistant to even powerful antimicrobial agents.^10,11^ Current treatments, such as painful surgical debridement and prolonged antibiotic use, often have limited success and can contribute to antibiotic resistance.^12,13^ To address this challenge, researchers are exploring strategies to prevent biofilm formation. This approach holds promise for reducing the need for invasive procedures and combating infections more effectively.^14^

**Fig. 1:**
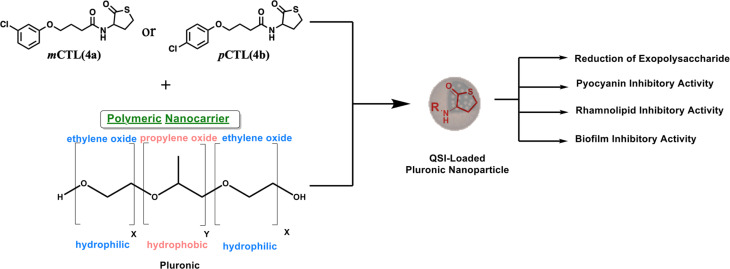
Illustration of the work design.

*P. aeruginosa* uses the quorum sensing (QS) system for cells to communicate and generate biofilm layers.^15^ This system depends on the surrounding bacterial population. Through QS, bacteria can release and detect chemical signaling molecules known as autoinducers. These autoinducers regulate gene expression, influencing processes such as toxin production, biofilm formation, bioluminescence, swarming motility, sporulation, and horizontal gene transfer.^16^ There are three main QS systems in *P. aeruginosa*. Two of these systems, *las* and *rhl*,^17^ utilize *N*-acyl homoserine lactones (AHLs) as signaling molecules. As the bacterial population increases, the specific autoinducers *N*-(3-oxododecanoyl)-l-homoserine lactone (OdDHL) and *N*-butyryl l-homoserine lactone (BHL) build up (Fig. 1). Once they reach a high enough level, these autoinducers can bind to special proteins known as *lasR* and *RhlR*. This binding activates genes that control the colonial behavior of bacterial cells, including biofilm formation. Chemically, pyocyanin and rhamnolipids are key virulence factors of *P. aeruginosa* and are regulated through its interconnected QS systems.^18^ Pyocyanin production is tightly controlled by *Las* and *Rhl* to disrupt the host cell function and immune responses. Simultaneously, rhamnolipids, governed by the *Rhl* system, contribute to biofilm development and enhancing bacterial motility. By disrupting bacterial communication, scientists aim to weaken their defenses and enhance antibiotic effectiveness, potentially slowing antibiotic resistance.^19^ In this regard, *meta*-chlorothiolactone (*m*CTL, 4a) and *para*-chlorothiolactone (*p*CTL, 4b) have been reported to inhibit the *LasR* protein, leading to biofilm disruption (Fig. 1).^20^

However, the inhibitory effects of *m*CTL and *p*CTL against *LasR* have also been reported;^13,20^ the hydrophilic nature of *m*CTL and *p*CTL (*c* Log *P* of both is 2.9) limits their ability to pass, in sufficient concentration, through the biofilm layers, which are known to have a lipophilic matrix. To facilitate the penetration of 4a and 4b through the hydrophobic layers of pseudomonal biofilms, both compounds were encapsulated inside nanoparticles (NPs). NPs offer a promising platform for developing effective antimicrobial agents. They enhance drug stability, allow sustained release, and improve biofilm penetration. In contrast, other widely studied strategies, such as the use of antimicrobial peptides (AMPs), face some challenges, like enzymatic degradation and high production costs.^21^ Our nanoparticle formula provides a more stable and cost-effective alternative with a strong biofilm inhibition potential.^22^

In this work, pluronic copolymers were selected to build 4a- and 4b-loaded NPs because pluronic copolymers boast several advantages for drug-delivery applications.^23^ Their biocompatible and non-toxic nature minimizes potential side effects,^24^ while their commercial availability and economical cost make them a practical choice for large-scale production.^25^ This study aimed to develop NPs with optimal physicochemical properties for the delivery of 4a and 4b (Fig. 1) as a novel strategy to combat *P. aeruginosa* by inhibiting biofilm formation and pyocyanin production. Microbiological investigations were performed and revealed that the nanoformulated forms of both molecules exhibited remarkable antibiofilm and antivirulence properties against *P. aeruginosa* with a prolonged release profile.

## Results and discussion

### Small molecule synthesis and nanoformula preparation

**Scheme 1:**
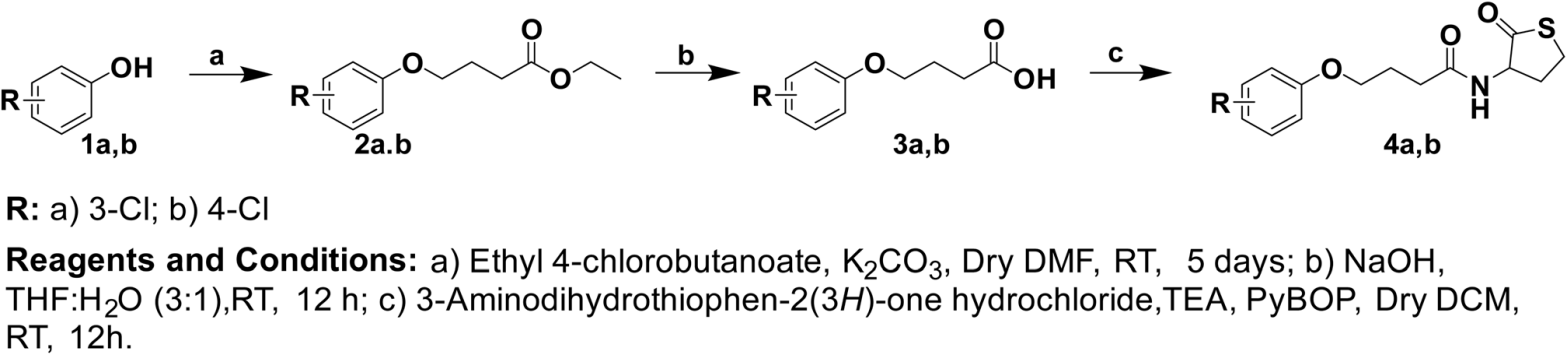
Preparation of compounds 4a and 4b.

The designated small molecules were synthesized as reported in an earlier study.^20^Scheme 1 shows the detailed conditions used to synthesize both molecules 4a and 4b.

Briefly, the appropriate phenol derivatives were allowed to react with ethyl 4-chlorobutanoate under basic conditions followed by saponification and insertion of the thiolactone head through the ordinary amide bond formation condition.

The CTL-loaded NPs were prepared using nanoprecipitation techniques. This method of preparation is particularly suitable for the encapsulation of hydrophobic compounds. The nanoencapsulation of lipophilic materials enhances their bioavailability.^26^ Pluronic copolymers were selected to construct the *m*CTL and *p*CTL-loaded NPs, because they offer several advantages for drug-delivery applications.

### Characterization of CTL-loaded NPs

**Fig. 2:**
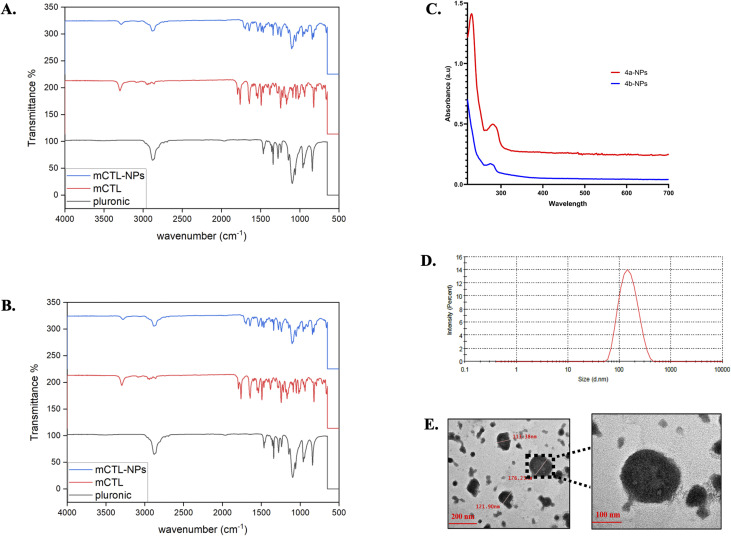
FTIR spectra of (A) PLF-108 (black), 4a (red) and 4a-NPs (blue); (B) PLF-108 (black), 4b (red), and 4b-NPs (blue); (C) UV-vis spectra of 4a-NPs (red) and 4b-NP (blue); (D and E) Particle size and TEM micrographs of 4a-NPs.

To characterize our nanoformulas, Fourier transform infrared (FTIR), dynamic light scattering (DLS), and transmission electron microscopy (TEM) analysis were employed. The spectra of 4a-NPs and 4b-NPs were compared with their free forms and the PLF-108 nanoparticles, as shown in Fig. 2A and B, respectively. Both 4a and 4b exhibited characteristic NH stretching peaks at 3267, 2941 (CH Aliphatic), 1743 (C=O stretching), and 1457 cm^−1^ (CH_2_ bending) (Fig. 2A and B (red)). On the other hand, peaks were observed at 2888 (C–H bending), 1467 (CH_2_ bending), and 1108 cm^−1^ (C–O–C stretching) in PLF-108 (Fig. 2A and B (black)). Characteristic peaks of 4a, 4b and the PLF-108 nanoparticles were typically found in the spectra of the CTL nanoparticles (Fig. 2A and B (blue)), indicating there was no chemical interaction between the CTL and pluronic matrix. In addition, the peaks in the range of 500–1500 cm^−1^ of the CTL nanoparticles were relatively increased compared to in the spectra of CTL and the PLF-108. The formation of our NPs was also confirmed utilizing UV-visible spectroscopy (Fig. 2C). Significant surface plasmon resonance peaks were observed at 279 nm. These results indicated that CTL may be encapsulated into the PLF-108 nanoparticles.

Further, the particle size and TEM analysis of the 4b-loaded NPs, as a representative of our work, was employed. The 4b-loaded NPs size was represented as the intensity (%) and the diameter was 158 nm (PDI: 0.140) with a zeta potential of−11.8 mv, see Fig. 2D. In addition, TEM micrographs of the developed nanomicelles were recorded and demonstrated the successful preparation of spherical CTL-NPs with sizes ranging from 100 to 180 nm, see Fig. 2E.

### Encapsulation efficacy and *in vitro* cumulative release studies

**Fig. 3:**
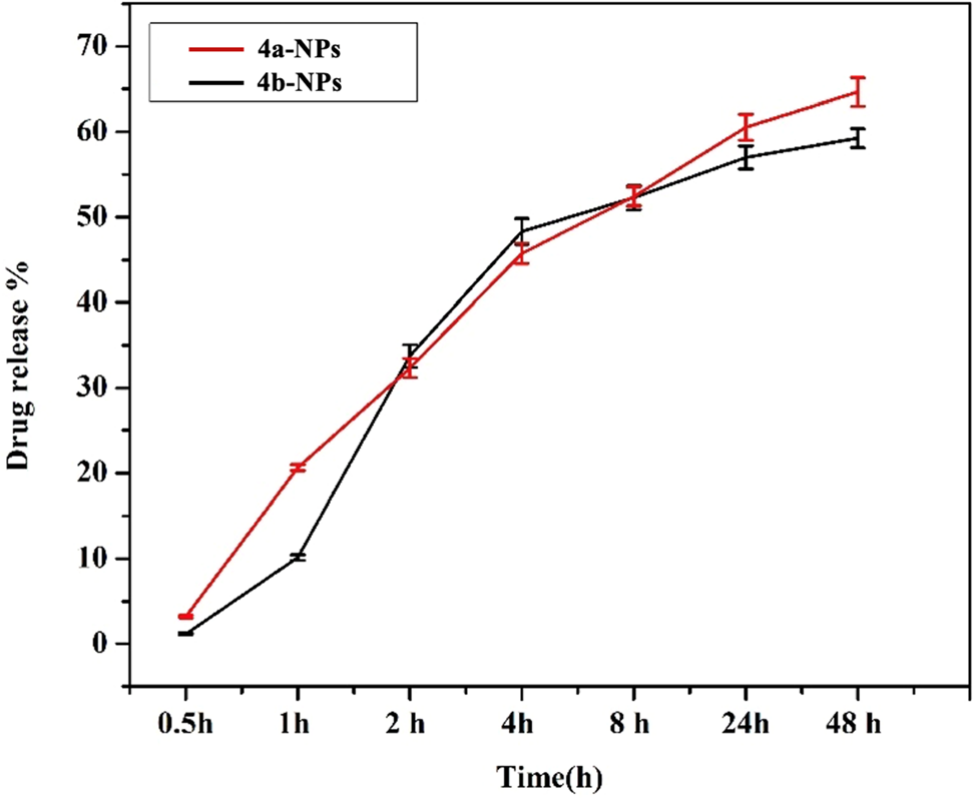
Cumulative release profiles of 4a (red) and 4b (black) from the prepared pluronic nanomicelles at 37 °C in PBS, pH 7.4.

Next, the encapsulation of 4a and 4b in pluronic PLF-108 was determined by measuring the encapsulation efficiency (EE%). The data showed that the encapsulation of both nanoparticles was 88.50% and 83.35% respectively. The cumulative release profiles of the loaded *p*CTL and *m*CTL were evaluated over 24 and 48 h in PBS at pH 7.4 (Fig. 3). The release profiles of pCTL and mCTL were obtained by plotting the cumulative percentage release of *p*CTL and *m*CTL *versus* time until attaining almost a constant value after 48 h. The percentage release of *p*CTL and *m*CTL from the pluronic NPs showed higher cumulative releases over 24 h (57% and 60.5%, respectively). This was followed by a gradual increase in the release patterns to 59.26% and 64.66% within 48 h. The release profiles of 4a and 4b from the loaded pluronic NPs were in accordance with them overcoming their entrapment within the polymeric hydrophobic core.

## Microbiological evaluation

### Antimicrobial screening of the synthesized compounds against *Pseudomonas aeruginosa*

Initially, we screened the synthesized compounds (4a, 4b) and their nanoparticle forms against *P. aeruginosa* ATCC 25668 and ATCC 27583, as assessed by the minimum inhibitory concentration (MIC) method using the resazurin based-broth microdilution method using polystyrene microtiter plates with 96 wells. None of the tested molecules demonstrated any meaningful activity against the two *P. aeruginosa* strains (Table S1 and Fig. S1†). This finding was consistent with previous reports. Based on these results, we selected sub-MIC concentrations to further investigate the potential of these compounds to inhibit biofilm formation, where non-bactericidal mechanisms may contribute to antibiofilm activity.

### Effect on *P. aeruginosa* biofilm

**Fig. 4:**
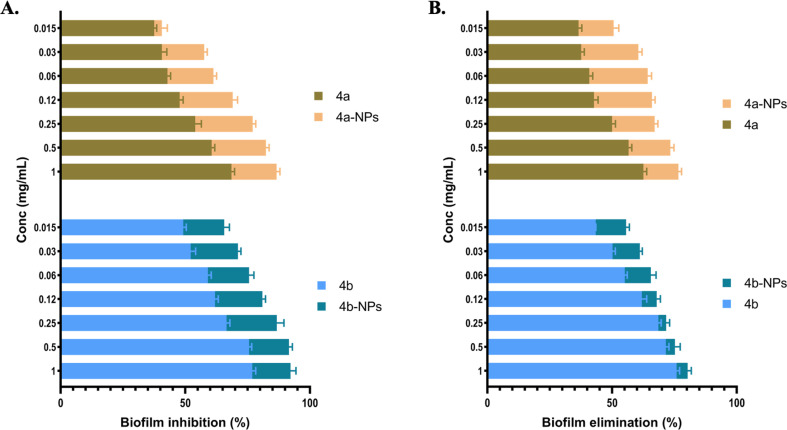
Effect of 4a-NPs, 4a, 4b-NPs, and 4b on the formation (A) and destruction of preformed (B) *P. aeruginosa* biofilms.

Infections caused by biofilm-forming bacteria, including *P. aeruginosa*, are notoriously difficult to treat due to adaptive multidrug resistance.^27^ Conventional antibiotics may fail to eliminate biofilms for a variety of reasons. Antibiotic resistance is multidimensional, comprising biofilm formation, adaptive stress responses, and metabolic inactivation due to nutritional and gas constraints.^28^ Thus, treating microbial biofilms with standard antibiotics becomes difficult. As a result, the use of new antibiofilm agents has attracted increasing attention to inhibit biofilm development. The present investigation aimed to investigate the impact of 4a, 4b, and their nanoformulas, *m*CTL-NPs and *p*CTL-NPs, on monoculture biofilm formation by *P. aeruginosa*. First, the biofilm eradication activity of our compounds was studied using crystal violet (CV) methods at a range of concentrations of 1, 0.5, 0.25, 0.12, 0.06, 0.03 and 0.015 mg mL^−1^. Briefly, *P. aeruginosa* (ATCC 25668) was inoculated at about 10^5^ CFU mL^−1^ in 96-well plates and incubated for 24 h at 37 °C. Following, the biofilm formation was quantified by CV staining, which provided visual evidence of the inhibitory activity of these compounds. The data and observations from this assay are presented in Fig. 4A. We found that all the tested compounds exhibited biofilm formation inhibition in a dose-dependent manner. In addition, the nanoformulated compounds demonstrated superior efficacy compared to the free compounds at all tested concentrations. For instance, the highest biofilm inhibition was recorded by 4a-NPs and 4b-NPs at a concentration of 1 mg mL^−1^ with 86.69% and 92.20% inhibition, respectively. These values were ∼15% higher than the free forms of 4a and 4b, indicating the effectiveness of our nanoformulas over the free drugs. At the lowest used concentration, 0.015 mg mL^−1^, 4b-NPs still demonstrated promising activity against *P. aeruginosa* biofilm with an inhibition % of ∼70%.

There are two major obstacles that hinder the inactivation of mature biofilms. First, the extracellular polymeric matrix (EPS), which shields bacteria from physical impacts and the penetration of large molecules, making microbes within the biofilm up to 1000 times more resistant than individual cells. Second, alterations in the gene expression of bacteria can enhance their resistance to various disinfectants and antibiotics. Therefore, the inactivation of preformed biofilms is one of the most promising approaches for the prevention of bacterial infection and control of antimicrobial resistance.^29^

Next, we tested our molecules against the preformed *P. aeruginosa* biofilm, as shown in (Fig. 4B). After incubation of the pathogens in wells to form a biofilm for 24 h, different concentrations of these compounds were added at the same previously used concentrations to each well, followed by further incubation at 37 °C for another 24 h. Both 4a-NPs and 4b-NPs kept the highest elimination power against the preformed *P. aeruginosa* biofilm, with inhibition % ranging from 75% to 50% and 80.3% to 55.6%, respectively. These values were obviously higher than the free molecules 4a and 4b, especially at the lower concentrations. This observation may be due to the negative surface charge of the prepared nanoparticles, which facilitated the uptake of these NPs by the bacteria.^30^

**Fig. 5:**
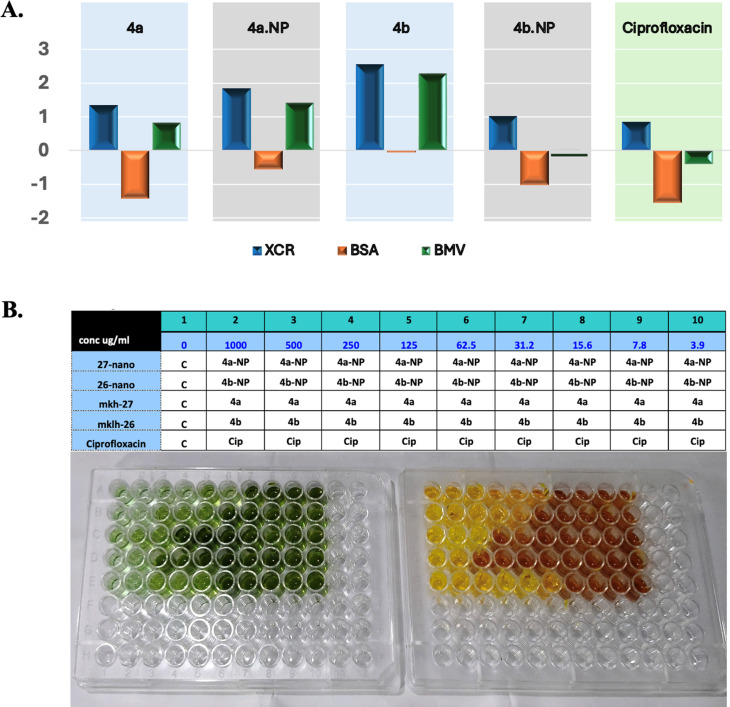
(A) Biofilm kinetics compared to ciprofloxacin. Bars represent (blue) XCR (XTTN/CVN, indicating metabolic activity relative to biomass), (orange) BSA ((XTTN/CVN) – [(CVN + XTTN)/2], a weighted descriptor of metabolic activity and biomass), and (green) BMV (XTTN – CVN, combining biofilm activity and biomass). Data were derived from normalized crystal violet (CVN) and XTT (XTTN) assays at a treatment concentration of 1 mg mL^−1^. Higher XCR and BMV values indicate greater biofilm inhibition, while lower BSA values reflect a balanced inhibition of metabolic activity and biomass; (B) experiment design and representative images of CV-stained (left) and XTT-stained (right) biofilm assay plates.

Next, the biofilm biomass and its metabolic activity after incubation with our treatments and in comparison with ciprofloxacin was measured at nine serial dilutions starting at 1 mg mL^−1^ (Fig. 5B). The formed biofilms were determined using CV whereas the activity was assessed with the XTT. In this assay, three indexes were measured, BSA which indicates the specific activity of the biofilm, BMV, which is a complex score of the biofilm ability to grow and be active, and XCR, a parameter used to measure the metabolic activity of a biofilm relative to its biomass.^31^ Data from this assay are summarized in Fig. 5. Our nanoformulas showed enhanced biofilm inhibition compared to their native congeners, as indicated by the BSA, BMV, and XCR metrics. Compound 4b-NP exhibited a higher XCR (1.844) and BMV (1.427) compared to 4b (XCR = 1.349, BMV = 0.825). These high values indicated an improvement of metabolic activity disruption and biomass reduction. Additionally, 4a-NP demonstrated a slight improvement over 4a, with an XCR value of 1.012 *versus* 2.558 and BMV of 0.023 *versus* 2.288 (Fig. 5A). It was surprising that at the highest concentration (1 mg mL^−1^), the reference molecule (ciprofloxacin) was outperformed by our nanoformulas (Fig. 5B).

The above findings suggest that our nanoformulas may be employed as superior antibiofilm agents against *P. aeruginosa*. They also highlight that the biofilms of *P. aeruginosa* exhibited some resistance against 4a and 4b treatment. This resistance could be attributed to its viscous extracellular matrix that limits their access.^29^ The marked elimination of the preformed biofilm by the nanomicelles suggested the capability of these compounds to penetrate the EPS matrix and lipid bilayers of microbial cells by exerting effects on the bacteria, thereby diminishing their protective biofilm layer.

Our findings suggest that the nanoparticle formulations of 4a and 4b showed enhanced biofilm disruption, likely due to the improved penetration and sustained release of the small molecules within the biofilm matrix. It was noteworthy that the physicochemical characteristics of the NPs, including their small size and surface charge, could also play a role in facilitating interactions with the negatively charged components of the EPS.^32–34^ This interaction my promote a deeper infiltration and localized delivery of the compounds into the biofilm matrix. The concise molecular targets of our formula though remain to be elucidated. Nevertheless, it is plausible that the small molecules interfere with bacterial metabolic pathways or EPS biosynthesis, which leads to weakening the biofilm structural integrity and reducing its biomass. Further studies are warranted to point out the precise biochemical interactions and confirm the pathways involved in such a biofilm inhibition mechanism.

### Pyocyanin–rhamnolipid inhibitory activity

**Fig. 6:**
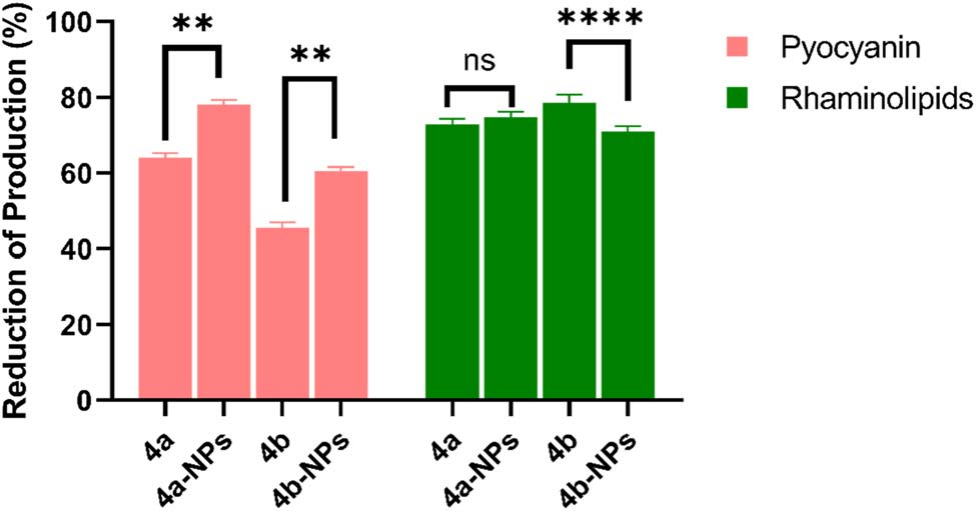
Effect of 4a, 4a-NPs, 4b and 4b-NPs on the production of pyocyanin and rhamnolipid. ** and **** correspond to *p* < 0.002 and *p* < 0.0001, respectively, as analyzed by one sample *t* test, and ns indicates no significant statistical difference between the tested concentrations.

It was reported that attenuation of the virulence characteristics combined with the prevention of *P. aeruginosa* biofilm formation is considered a promising therapeutic strategy.^29^ Moreover, targeting these virulence factors makes it possible to utilize conventional antibiotics and lower the resistance mechanisms.^33,34^ We measured the ability of our molecules to inhibit pyocyanin and rhamnolipid production using established extraction and spectrophotometric methods, as reported previously.^35,36^ Briefly, pyocyanin was extracted from treated culture supernatants with chloroform and acidified before measuring the absorbance at 390 nm.^37^ Meanwhile, rhamnolipids were quantified by acidifying the treated supernatant and measuring the absorbance at 570 nm. The data from this assay are summarized in Fig. 6. It was found that 4b-NPs, 4b, 4a-NPs, 4a reduced the production of pyocyanin by 60.4%, 45.6%, 78.2%, and 64%, respectively. In the study by O'Loughlin *et al.*,^20^4a showed a potent inhibition of pyocyanin production by 50% at a concentration of 9 μM. Meanwhile, the synthetic compound 4-aminopyridine was found to reduce the production of pyocyanin by 70% in a study by Miller *et al.*^39^

The reduction of rhamnolipid production was observed (Fig. 6). The maximum reduction was achieved by 4b-NPs at 78%, while a lower reduction percentage of 71.6% was observed with compound 4b.

The effect of the CTLs against virulence factors may be due to their interference with the signaling pathways through their binding to essential components of quorum sensing, hindering the coordination of bacterial actions, such as the generation of virulence factors and biofilm formation.^40^ Moreover, it was demonstrated that the nanoparticles enhanced the inhibitory effect on pyocyanin production compared to their free counterparts. This suggested that the encapsulation of 4b and 4a within the nanoparticles improved their ability to target and suppress the production of pyocyanin by *P. aeruginosa*.

### Quantification of exopolysaccharide (EPS)

**Fig. 7:**
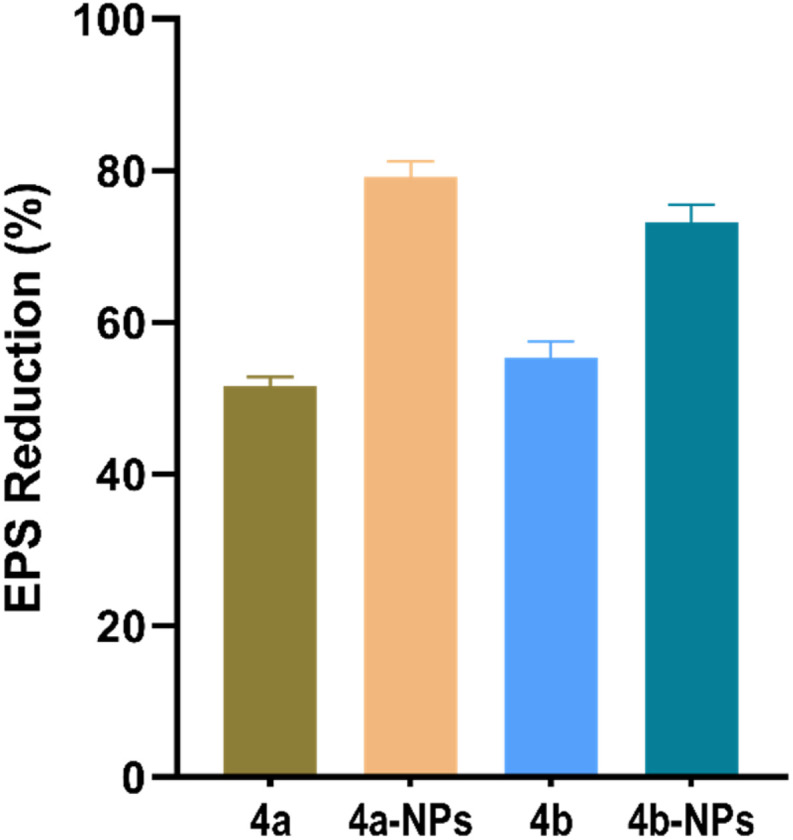
Inhibition of the exopolysaccharide of *P. aeruginosa* by the effects of 4a, 4a-NPs, 4b and 4b-NPs.

The biofilm architecture of *P. aeruginosa* is highly complex, comprising numerous types of components, and is one of the principal barriers to several antimicrobial treatments.^33^ Apart from the structural component, a self-secreted exopolymeric substance (EPS) surrounds the bacterial population, which is mainly composed of proteins, polysaccharides, lipids, DNA, and other macromolecules.^32^ The EPS is critical for biofilm formation and provides an effective barrier to the penetration of antibiotics.^33^ Moreover, the EPS allows cells immobilization, microconsortia formation and cell-to-cell communication.^36^ In this study, we investigated the EPS production of *P. aeruginosa* treated with 4a, 4a-NPs, 4b and 4b-NPs by quantifying its production using a phenol/sulfuric acid colorimetric assay.^41^ This method can reliably reflect the reduction in EPS production upon treatment. Briefly, bacterial cultures treated with the compounds were incubated, and EPS was extracted and precipitated using ethanol. The precipitated EPS was then allowed to react with a mixture of phenol and sulfuric acid to develop a measurable color change. The results (Fig. 7) show there were significant decreases in EPS production by 79.17%, 73.17%, 55.3% and 51.5% by the effect of 4a-NPs, 4b-NPs, 4b and 4a, respectively.

These significant inhibitions in EPS production correlated with the previously observed reduction in biofilm biomass and viability (Fig. 4) and suggest that the inhibition of EPS synthesis is a key mechanism by which these compounds disrupt biofilm formation. The enhanced EPS inhibition by the nanoparticle formulations likely contributed to their superior antibiofilm efficacy compared to the native compounds. Together, these findings underscore the role of EPS disruption in weakening the biofilm structural integrity and support the potential of nanoparticle-based delivery systems to improve therapeutic outcomes.

### Cytotoxicity investigations

To examine the tolerability of our nanoformulas, we evaluated their toxicity *in vitro* against human diploid (WI-38) cells using the MTT assay. Our nanoparticles were evaluated at five concentrations in comparison with the corresponding small molecules and ciprofloxacin. The IC_50_ values of the tested compounds are summarized in Table 1. Notably, 4b exhibited the highest cytotoxicity with an IC_50_ of 150.6 μM, followed by ciprofloxacin (186.67 μM) and 4a (266.42 μM). Both 4a-NP and 4b-NP showed low to moderate cytotoxicity, with IC_50_ values of 368 and 248 mM, respectively. These findings indicate that the encapsulation of 4a and 4b into nanoparticles not only improved their antimicrobial profile but also improved their biocompatibility.

**Table 1:** IC_50_ of 4a-NP and 4a-NP against WI-38 cell line in comparison with 4a and 4b and ciprofloxacin

Compound	IC_50_ (μM)
4a-NP	368.03 ± 12.1
4b-NP	248.75 ± 8.18
4a	266.42 ± 8.76
4b	150.6 ± 4.95
Cip	186.67 ± 6.14

## Conclusion

The rise of antibiotic resistance poses a significant global health challenge, necessitating innovative strategies to combat persistent bacterial infections. In this study, we synthesized two CTL derivatives, *m*CTL (4a) and *p*CTL (4b), and encapsulated them in pluronic nanoparticles using the nanoprecipitation method. Comprehensive characterization of the nanoformulations, including by FTIR, DLS, TEM, and UV-visible spectroscopy, confirmed the successful encapsulation of 4a and 4b within the nanoparticles, with particle sizes ranging from 100 to 180 nm and encapsulation efficiencies of 88% and 83%, respectively. Our nanoformulations demonstrated sustained release profiles, with cumulative releases of 59% and 64% over 48 h, indicating their potential for prolonged therapeutic action.

Microbiological evaluation revealed that while the free compounds (4a and 4b) lacked significant antimicrobial activity against *P. aeruginosa*, their nanoformulated forms exhibited remarkable antibiofilm and antivirulence properties. The nanoformulations (4a-NPs and 4b-NPs) achieved up to a 92% inhibition of biofilm formation and eradicated preformed biofilms by up to 80%, outperforming their free forms by approximately 15%. We argue that this enhanced efficacy is related to the nanoparticles' ability to penetrate the extracellular polymeric matrix (EPS) and disrupt biofilm integrity. Furthermore, the nanoformulations significantly reduced the production of key virulence factors, including pyocyanin and rhamnolipids (by up to 78%), as well as decreased EPS production by up to 79%. These observations highlight the multifaceted mechanism of action of these nanoformulations. In addition, cytotoxicity investigations indicated that the nanoformulations could improve the biocompatibility of the targeted compounds and potentially enhance their therapeutic safety profile.

Collectively, these findings underscore the potential of 4a-NPs and 4b-NPs as a promising approach for treating *P. aeruginosa* infections. By targeting biofilm formation, virulence factor production, and EPS secretion, these nanoformulations offer a robust strategy to combat antibiotic resistance. The antimicrobial nanoformulations described in this work offer a novel strategy for the potential treatment of *P. aeruginosa* infections through targeting and reducing the expression of virulence factors involved in chronic infection, rather than directly inhibiting bacterial growth.^42^ Future studies will focus on optimizing these formulations and advance their development as next-generation antibiofilm agents.

This work offers a novel approach by formulating reported antimicrobial compounds into nanoparticles that specifically target the virulence mechanisms of *P. aeruginosa* rather than directly killing the bacteria. These mechanisms include biofilm formation, pyocyanin, rhamnolipids, and EPS production. Such a strategy aims to reduce the pathogenicity and persistence of the targeted bacteria while potentially lowering the risk of resistance development. The superior inhibitory effect observed with our nano-encapsulated compounds highlights the advantage of nanoparticle delivery systems in improving drugs penetration into targets and antivirulence activity.

## Experimental section

### Chemicals

Pluronic F-108 (PLF-108) was obtained from Aldrich. Acetone, chloroform and ethanol were obtained as analytical grade from Analar and used as received. Tryptic soya broth was purchased from Oxoid (0 M0129).

### Preparation of CTLs-loaded pluronic nanoparticles

**Fig. 8:**
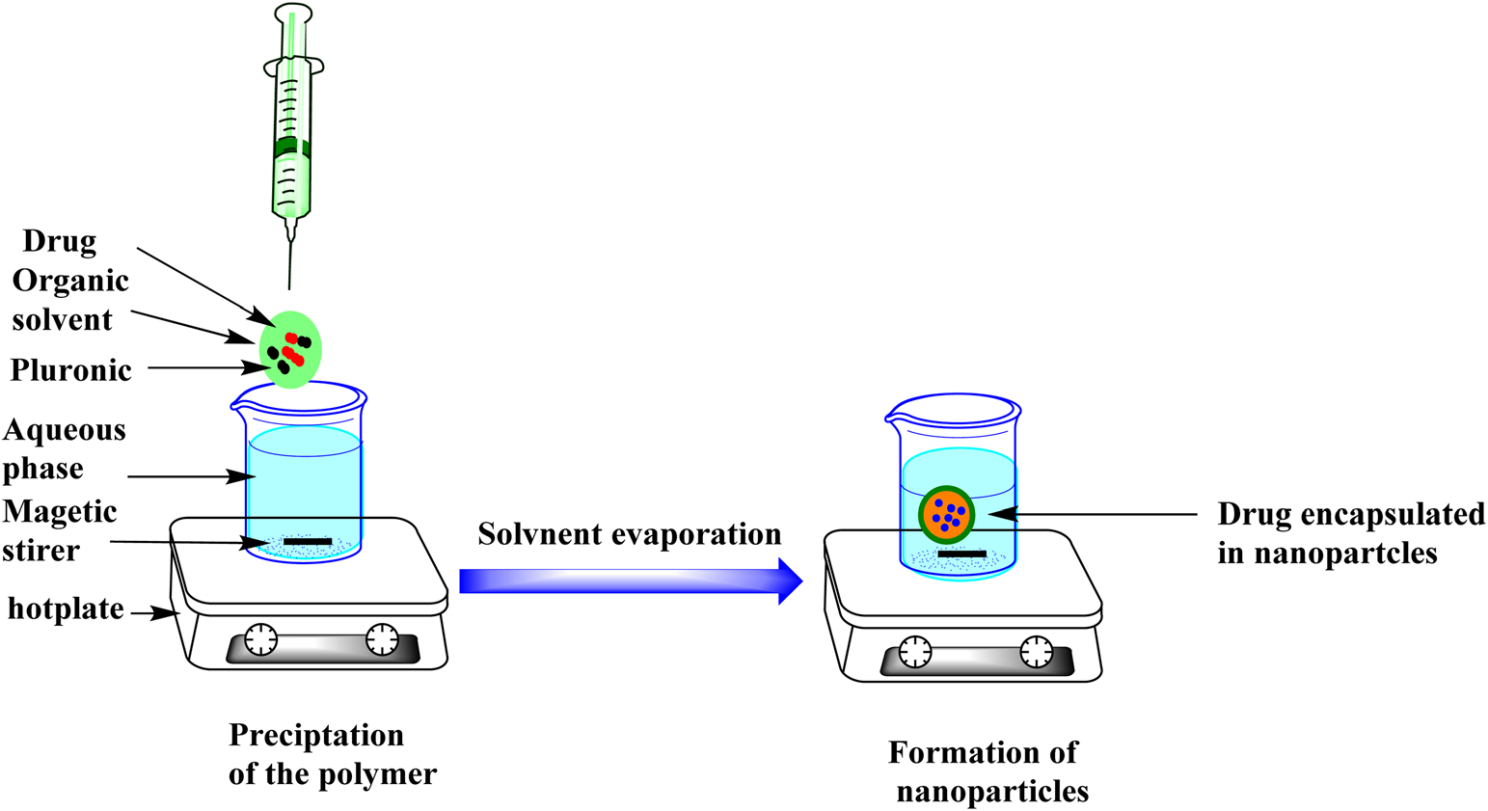
Nanoprecipitation technique.

CTL-loaded pluronic nanoparticles (CTL-NPs) were prepared using a nanoprecipitation method.^23^ This method involved a specific ratio of polymer to CTL of 2 : 1. In brief, about 250 mg of pluronic F-108 and 125 mg of CTL were dissolved together in 30 mL of 95% acetone to form the organic phase. After complete dissolution, this organic phase was slowly added, with continuous stirring, to 80 mL of distilled water (Fig. 8). The final suspension was left to allow evaporation of the acetone. Finally, this concentrated nanosuspension was freeze-dried using a Labconco freeze dryer.

### Physicochemical characterization of the prepared nanoparticles

#### Measurement of the nanoparticle size and zeta potential

The average particle size of the prepared nanoparticles, polydispersity (PDI) index and surface charge were determined using a zetasizer (Nano-ZS, Malvern, UK). Measurements were performed in aqueous-diluted samples (2 : 1 ratio) at 25 °C.

### Transmission electron microscopy (TEM)

Transmission electron microscopy (TEM) was used to evaluate the nanoparticles morphology. The material suspension was subjected to ultrasound then a droplet of particles was placed on a copper grid. After being dried, a small amount of phosphotungestic acid (1%) was added until it dried and it was then tested by TEM (Tokyo, Japan, JEM-2100, JEOL).

### Fourier transform infrared spectroscopy (FTIR)

IR spectroscopy was performed using a Fourier transform infrared spectroscopy instrument (FTIR, NICOLET, iS10, Thermo Scientific). The wavelength range for the IR scan was 4000–600 cm^−1^.

### Determination of the encapsulation efficiency (EE%)

The encapsulation efficiency (EE%) of the CTL-loaded pluronic nanoparticles formulation was determined by an indirect method. After preparing fresh CTL-loaded pluronic NPs, they were separated from the aqueous medium containing the free CTL in supernatant by centrifugation at 14 000 rpm for 30 min at 4 °C. After a suitable dilution with distilled water, the amount of free CTL in the supernatant was analyzed by UV-visible spectrophotometry (Biochrome libra S22, Thermo-Fisher Scientific, Waltham MA, USA).

The encapsulation efficiency was determined as follows:1
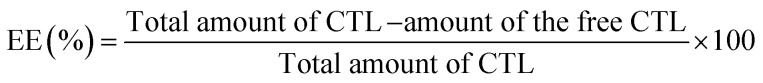


### *In vitro* release study

An *in vitro* release study of the CTL from the prepared CTL-NPs was performed at pH 7 in phosphate buffer solution (PBS) using the dialysis method [21]. In brief, 1 mL CTL-NPs (containing 4 mg mL^−1^ of CTL) was transferred to a dialysis bag (spectra Por7, 10 kD), which was then tied at the end and immersed in a cell containing PBS (1 mL) in a dialysis membrane. Then the dialysis membrane was suspended in a 50 mL beaker containing 30 PBS (pH 7.4). The test was performed using a shaker water bath at 100 rpm at 37 ± 0.5 °C. The samples were withdrawn at pre-determined time points and replaced by fresh PBS solution. The collected samples were measured by UV-vis (Evolution UV 600, Thermo Scientific, USA). Finally, the released amount of CTL was calculated and then a cumulative release curve was drawn.

### Inhibition of biofilm formation by the CTL-loaded pluronic nanoparticles

For the assessment of the inhibition of biofilm formation, bacteria cultures (*P. aeruginosa* ATCC 25668) were recovered from frozen stocks kept at −80 °C, inoculated into tryptic soy broth (TSB), and incubated under 37 °C for 24 h. The optical density (OD595 nm) of the cultures was adjusted to 1.2 (∼1–5 × 10^9^ CFU mL^−1^) and then diluted with TSB to about 10^5^ CFU mL^−1^. Then, a 150 μL aliquot from the culture was transferred to a 96-tissue culture-treated Petri dish (TPP, Switzerland) to provide enough surface area for biofilm formation at 37 °C for 24 h. Then, 150 μL of *m*CTL, *m*CTL-NPs, *p*CTL or *p*CTL-NPs with concentrations of 1, 0.5, 0.25, 0.12, 0.06, 0.03 and 0.015 mg mL^−1^ were added to each well, while the *P. aeruginosa* biofilm was used as the control, followed by incubation at 37 °C for 24 h. The biofilms in the wells were stained by crystal violet (CV) staining. Briefly, the TSB medium was removed, and each well was rinsed twice with PBS (300 μL) to remove loosely attached bacteria. Then, the wells were left to air dry for 15 min, and then stained with 300 μL of 0.1% CV solution for 45 min at room temperature.^38^ Excess CV solution was removed, and the wells were washed three times with PBS followed by the addition of 300 μL of 95% ethanol to solubilize the crystal violet dye adhering to the biofilm. The liquid content of each well was transferred to a new flat-bottom microplate and the absorbance was measured at 595 nm by UV-vis (Evolution UV 600, Thermo Scientific, USA).^43^ The inhibition percentage was calculated with the following formula,2
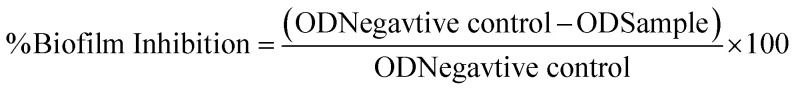


### Inactivation of a preformed biofilm by the CTL-loaded pluronic nanoparticles

For inactivation of the preformed biofilms, the bacterial cultures were inoculated into 96-well tissue culture plates. The plates were incubated for 24 h at 37 °C. Subsequently, the wells were gently rinsed with 200 μL of PBS to remove any loosely attached bacteria. 4a, 4a-NPs, 4b or 4b-NPs were then added to each well to achieve final concentrations of 1, 0.5, 0.25, 0.12, 0.06, 0.03 and 0.015 mg mL^−1^, followed by an additional 24 h incubation at 37 °C.^44^ The biofilms were gently rinsed with PBS and quantified using CV staining as previously described. The elimination percentage was calculated using the following formula,3
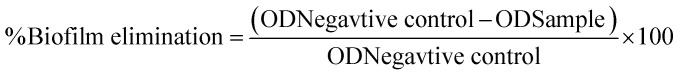


### Assays for the virulence factors of *P. aeruginosa*

The evaluation of the compounds against the virulence factors of *P. aeruginosa* was performed as previously described.^45^ An overnight culture of *P. aeruginosa* was placed in contact with *m*CTL, *m*CTL-NPs, *p*CTL or *p*CTL-NPs at concentration of 0.015 mg mL^−1^. *P. aeruginosa* without treatment was used as the control. After 24 h incubation, the cultures were centrifuged at 10 000 rpm for 10 min, and then the virulence factors were determined in cell-free supernatant fluid aliquots.

### Pyocyanin quantification assay

The determination of the pyocyanin production was performed using the method described previously. The pigment was extracted from 750 μL of the supernatant with 375 μL of chloroform, and then the supernatant was removed, and the organic layer (blue color) was acidified with 300 μL of 0.2 M HCl. The supernatant took on a pink coloration, and from this, 150 μL was taken, which was neutralized with 150 μL of a tris buffer at 200 mM to read the absorbance at 390 nm.

### Rhamnolipids quantitative assay

The culture was centrifuged at 10 000×*g* for 10 min, and then the supernatants were collected, acidified with HCl (to pH 2) and the absorbance was measured at 570 nm.

The percentage pyocyanin and rhamnolipids production was determined using the following equation,4
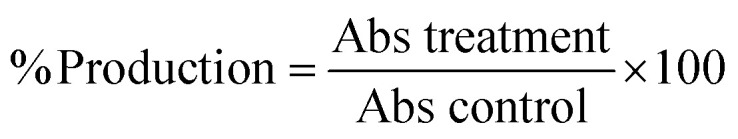


### Quantification of exopolysaccharide (EPS)

The extraction and quantification of polysaccharide were performed using the method of Singh *et al.* 2017.^46^ Briefly, the bacterial culture was grown with 4a, 4a-NPs, 4b or 4b-NPs at a concentration of 0.015 mg mL^−1^ in a 50 ml centrifuge tube. Bacterial culture without treatment was used as the negative control. The tubes were incubated for 24 h at 37 °C without shaking. After incubation, the cells were separated by centrifugation for 15 min at 10 000 rpm and the supernatant was discarded. The pellets were resuspended for 15 min in 50 ml high salt buffer and then centrifuged for 15 min at 10 000 rpm, and then an equal volume of ethanol was added to the collected supernatant and this was recentrifuged for 15 min at 10 000 rpm. Next, 1 ml from the precipitated EPS was mixed with 1 ml of cold 5% phenol and 5 ml of concentrated sulfuric acid to develop a red color. The intensity was measured at 490 nm.

### Cytotoxicity assay

Normal lung fibroblast (WI-38) cell lines (purchased from the American Type Culture Collection (ATCC), Manassas, VA, USA) were cultured in EMEM media containing 10% FBS, 1% NEAA, and 1% penicillin/streptomycin solution at 37 °C with 5% CO_2_. The cells were seeded at a density of 5000 cell per well in 96-well plates. Next, the cells were treated with the indicated concentrations. The plates were incubated for 24 h before the addition of 10 μL of reconstituted MTT (Sigma Chemicals), followed by incubation of the plates for 4 h. The samples' absorbance was determined with a 450 nm filter using the ROBONIK P2000 ELISA Reader.

## Abbreviations

CTL: chlorothiolactone
*m*CTL: *meta*-chlorothiolactone
*p*CTL: *para*-chlorothiolactone
*m*CTL-NPs: *meta*-chlorothiolactone nanoparticles
*p*CTL-NPs: *para*-chlorothiolactone nanoparticles
*P. aeruginosa*
: *Pseudomonas aeruginosa*
AMR: antimicrobial resistance
WHO: World Health Organization
EPS: exopolysaccharide
e-DNA: extracellular DNA
QS: quorum sensing
TEM: transmission electron microscopy

## Conflicts of interest

There are no conflicts to declare.

## Supplementary Material

RA-015-D5RA01831E-s001

## Data Availability

All data generated or analyzed during this study are included in this published article and its ESI files.†

## References

[cit1] Moradali M. F., Ghods S., Rehm B. H. (2017). Pseudomonas aeruginosa lifestyle: a paradigm for adaptation, survival, and persistence. Front. Cell. Infect. Microbiol..

[cit2] Mulani M. S., Kamble E. E., Kumkar S. N., Tawre M. S., Pardesi K. R. (2019). Emerging strategies to combat ESKAPE pathogens in the era of antimicrobial resistance: a review. Front. Microbiol..

[cit3] Balakrishnan V. S. (2022). WHO's first global infection prevention and control report. Lancet Infect. Dis..

[cit4] Pachori P., Gothalwal R., Gandhi P. (2019). Emergence of antibiotic resistance Pseudomonas aeruginosa in intensive care unit; a critical review. Genes Dis..

[cit5] Foucrier A., Dessalle T., Tuffet S., Federici L., Dahyot-Fizelier C., Barbier F., Pottecher J., Monsel A., Hissem T., Lefrant J. Y. (2023). Association between combination antibiotic therapy as opposed as monotherapy and outcomes of ICU patients with Pseudomonas aeruginosa ventilator-associated pneumonia: an ancillary study of the iDIAPASON trial. Crit. Care.

[cit6] Shi Q., Huang C., Xiao T., Wu Z., Xiao Y. (2019). A retrospective analysis of Pseudomonas aeruginosa bloodstream infections: prevalence, risk factors, and outcome in carbapenem-susceptible and-non-susceptible infections. Antimicrob. Resist. Infect. Control.

[cit7] Bongiovanni M., Barda B. (2023). Pseudomonas aeruginosa bloodstream infections in SARS-CoV-2 infected patients: a systematic review. J. Clin. Med..

[cit8] Reygaert W. C. (2018). An overview of the antimicrobial resistance mechanisms of bacteria. AIMS Microbiol..

[cit9] Thi M. T. T., Wibowo D., Rehm B. H. (2020). Pseudomonas aeruginosa biofilms. Int. J. Mol. Sci..

[cit10] Tuon F. F., Dantas L. R., Suss P. H., Tasca Ribeiro V. S. (2022). Pathogenesis of the Pseudomonas aeruginosa biofilm: a review. Pathogens.

[cit11] Karami P., Khaledi A., Mashoof R. Y., Yaghoobi M. H., Karami M., Dastan D., Alikhani M. Y. (2020). The correlation between biofilm formation capability and antibiotic resistance pattern in Pseudomonas aeruginosa. Gene Rep..

[cit12] Fernández-Billón M., Llambías-Cabot A. E., Jordana-Lluch E., Oliver A., Macià M. D. (2023). Mechanisms of antibiotic resistance in Pseudomonas aeruginosa biofilms. Biofilm.

[cit13] Mirghani R., Saba T., Khaliq H., Mitchell J., Do L., Chambi L., Diaz K., Kennedy T., Alkassab K., Huynh T. (2022). Biofilms: Formation, drug resistance and alternatives to conventional approaches. AIMS Microbiol..

[cit14] Kumar L., Bisen M., Harjai K., Chhibber S., Azizov S., Lalhlenmawia H., Kumar D. (2023). Advances in nanotechnology for biofilm inhibition. ACS omega.

[cit15] Miranda S. W. , Asfahl K. L., Dandekar A. A. and Greenberg E. P., Pseudomonas aeruginosa Quorum Sensing, in Pseudomonas aeruginosa: Biology, Pathogenesis and Control Strategies, Advances in Experimental Medicine and Biology, ed. A. Filloux and J. L. Ramos, Springer, Cham, 2022, vol. 1386, pp. 95–115 10.1007/978-3-031-08491-1_4 PMC9942581 36258070

[cit16] Patel K., Panchal R., Sakariya B., Gevariya M., Raiyani R., Soni R., Goswami D. (2024). Combatting Antibiotic Resistance by Exploring the Promise of Quorum Quenching in Targeting Bacterial Virulence. Microbeam.

[cit17] Ambreetha S., Singh V. (2023). Genetic and environmental determinants of surface adaptations in Pseudomonas aeruginosa. Microbiology.

[cit18] Lee J., Zhang L. (2015). The hierarchy quorum sensing network in Pseudomonas aeruginosa. Protein Cell.

[cit19] Choi H., Ham S.-Y., Cha E., Shin Y., Kim H.-S., Bang J. K., Son S.-H., Park H.-D., Byun Y. (2017). Structure–activity relationships of 6-and 8-gingerol analogs as anti-biofilm agents. J. Med. Chem..

[cit20] O'Loughlin C. T., Miller L. C., Siryaporn A., Drescher K., Semmelhack M. F., Bassler B. L. (2013). A quorum-sensing inhibitor blocks Pseudomonas aeruginosa virulence and biofilm formation. Proc. Natl. Acad. Sci. U. S. A..

[cit21] Saubenova M., Rapoport A., Yermekbay Z., Oleinikova Y. (2025). Antimicrobial Peptides, Their Production, and Potential in the Fight Against Antibiotic-Resistant Pathogens. Fermentation.

[cit22] Kumar L., Bisen M., Harjai K., Chhibber S., Azizov S., Lalhlenmawia H., Kumar D. (2023). Advances in nanotechnology for biofilm inhibition. ACS omega.

[cit23] Batrakova E. V., Kabanov A. V. (2008). Pluronic block copolymers: evolution of drug delivery concept from inert nanocarriers to biological response modifiers. J. Controlled Release.

[cit24] Thang N. H., Chien T. B., Cuong D. X. (2023). Polymer-based hydrogels applied in drug delivery: An overview. Gels.

[cit25] Bodratti A. M., Alexandridis P. (2018). Formulation of poloxamers for drug delivery. J. Funct. Biomater..

[cit26] El-Far Y. M., Zakaria M. M., Gabr M. M., El Gayar A. M., El-Sherbiny I. M., Eissa L. A. (2016). A newly developed silymarin nanoformulation as a potential antidiabetic agent in experimental diabetes. Nanomedicine.

[cit27] Novick R. P. (2003). Autoinduction and signal transduction in the regulation of staphylococcal virulence. Mol. Microbiol..

[cit28] Boles B. R., Horswill A. R. (2008). Agr-mediated dispersal of Staphylococcus aureus biofilms. PLoS Pathog..

[cit29] Høiby N., Bjarnsholt T., Givskov M., Molin S., Ciofu O. (2010). Antibiotic resistance of bacterial biofilms. Int. J. Antimicrob. Agents.

[cit30] Cui X., You J., Sun L., Yang X., Zhang T., Huang K., Pan X., Zhang F., He Y., Yang H. (2016). Characterization of Pseudomonas aeruginosa phage C11 and identification of host genes required for virion maturation. Sci. Rep..

[cit31] Corte L., Casagrande Pierantoni D., Tascini C., Roscini L., Cardinali G. (2019). Biofilm specific activity: a measure to quantify microbial biofilm. Microorganisms.

[cit32] Harmsen M., Yang L., Pamp S. J., Tolker-Nielsen T. (2010). An update on Pseudomonas aeruginosa biofilm formation, tolerance, and dispersal. FEMS Immunol. Med. Microbiol..

[cit33] Huang Y., Guo X., Wu Y., Chen X., Feng L., Xie N., Shen G. (2024). Nanotechnology's frontier in combatting infectious and inflammatory diseases: prevention and treatment. Signal Transduction Targeted Ther..

[cit34] Allen R. C., Popat R., Diggle S. P., Brown S. P. (2014). Targeting virulence: can we make evolution-proof drugs?. Nature Reviews Microbiology.

[cit35] Flemming H.-C., Wingender J., Szewzyk U., Steinberg P., Rice S. A., Kjelleberg S. (2016). Biofilms: an emergent form of bacterial life. Nat. Rev. Microbiol..

[cit36] Ganesh P. S., Rai V. R. (2018). Attenuation of quorum-sensing-dependent virulence factors and biofilm formation by medicinal plants against antibiotic resistant Pseudomonas aeruginosa. J. Tradit. Complementary Med..

[cit37] Hernando-Amado S., Alcalde-Rico M., Gil-Gil T., Valverde J. R., Martínez J. L. (2020). Naringenin inhibition of the Pseudomonas aeruginosa quorum sensing response is based on its time-dependent competition with N-(3-Oxo-dodecanoyl)-L-homoserine lactone for LasR binding. Front. Mol. Biosci..

[cit38] Cheng T., Liang J., He J., Hu X., Ge Z., Liu J. (2017). A novel rhamnolipid-producing Pseudomonas aeruginosa ZS1 isolate derived from petroleum sludge suitable for bioremediation. AMB Express.

[cit39] Miller L. C., O'Loughlin C. T., Zhang Z., Siryaporn A., Silpe J. E., Bassler B. L., Semmelhack M. F. (2015). Development of potent inhibitors of pyocyanin production in Pseudomonas aeruginosa. J. med. Chem..

[cit40] Wu L., Luo Y. (2021). Bacterial quorum-sensing systems and their role in intestinal bacteria-host crosstalk. Front. microbiol..

[cit41] DuBois M., Gilles K. A., Hamilton J. K., Rebers P. A., Smith F. (1956). Colorimetric method for determination of sugars and related substances. Anal. Chem..

[cit42] Lu L., Yu T., Wang H., Zhu X., Liao L., Zhu J., Li M. (2025). Virulence arresting drugs discovery by strategies targeting bacterial virulence: mainly focusing on quorum-sensing interference and biofilm inhibition. J. Pharm. Anal..

[cit43] Djordjevic D., Wiedmann M., McLandsborough L. (2002). Microtiter plate assay for assessment of Listeria monocytogenes biofilm formation. Appl. Environ. Microbiol..

[cit44] Bai X., Liu D., Xu L., Tenguria S., Drolia R., Gallina N. L., Cox A. D., Koo O.-K., Bhunia A. K. (2021). Biofilm-isolated Listeria monocytogenes exhibits reduced systemic dissemination at the early (12–24 h) stage of infection in a mouse model. NPJ Biofilms Microbiomes.

[cit45] Filloux A. and Ramos J.-L., Pseudomonas Methods and Protocols, Springer, 2014 10.1007/978-1-4939-0473-0 24936603

[cit46] Singh V. K., Mishra A., Jha B. (2017). Anti-quorum sensing and anti-biofilm activity of Delftia tsuruhatensis extract by attenuating the quorum sensing-controlled virulence factor production in Pseudomonas aeruginosa. Front. Cell. Infect. Microbiol..

