# Supplementary material for: Development and evaluation of new nanoformulated thiolactone derivatives for enhanced disruption of pseudomonal biofilms
Source: RSC Adv. 2025 Jun 24;15(27):21369–80. doi: 10.1039/d5ra01831e (PMC12186023; doi:10.1039/d5ra01831e)

Supplementary Information (SI) for RSC Advances.
This journal is © The Royal Society of Chemistry 2025

Supporting information

Development and Evaluation of New Nanoformulated Thiolactone Derivatives for Enhanced Disruption of *Pseudomonal* Biofilms

Mohamed K. Gaballah[1], Shaimaa A. Khalid[2,3], Mohamed A. Seleem[1,4], Amr K. A. bass[5], Ibrahim M. El-Sherbiny[2,**] and Abdelrahman S. Mayhoub[1,*]

[1]Center of Certified Reference Materials (CRM), Zewail City of Science and Technology, 6th of October City, Giza, 11566, Egypt.

[2] Reference Lab for Safety Analysis of Food of Animal Origin, Animal Health Research Institute (AHRI), Agricultural Research Center (ARC), Dokki, Egypt.

[3]Nanomedicine Laboratories, Center for Materials Science, Zewail City of Science and Technology, 6th October City, 12578 Giza, Egypt.

[4]Department of Pharmaceutical Organic Chemistry, Faculty of Pharmacy, Al-Azhar University, Cairo 11884, Egypt.

[5]Department of Pharmaceutical Chemistry, Faculty of Pharmacy, Minufia University, Minufia, Egypt.

[*]**Corresponding Author**: E-mail address: amayhoub@zewailcity.edu.eg; Ahmed Zewail Street, Zewail City of Science and Technology, October Gardens, 6th of October City, Giza, Egypt.

[**]**Co-corresponding Author**: E-mail address: ielsherbiny@zewailcity.edu.eg; Ahmed Zewail Street, Zewail City of Science and Technology, October Gardens, 6th of October City, Giza, Egypt.

Antimicrobial Screening of the synthesized compounds against *Pseudomonas aeruginosa*

In this experimental work, the possible antimicrobial activities of the chemical compounds that encoded (26, 26N, 27 and 27N) were tested against standard strains (*Pseudomonas aeruginosa* ATCC 25668 and ATCC 27583) was assessed by Minimum inhibitory concentration (MIC) method using resazurin based-broth microdilution method by polystyrene microtiter plates 96 wells as indicated by the USA Clinical and Laboratory Standards Institute. A pure 0.5 McFarland standard of each tested isolate suspended in tryptic soya broth (TSB) was prepared. One hundred microliters of each tested compound (4a, 4a-NPs, 4b, 4b-NPs) stock solution of concentration 1024 µg/ml in 20% DMSO, were added to the 1st well of each raw and TSB was inoculated as 50 µL/well into the remaining wells. Fifty microliters of each stock solution in the 1st well were transferred into the 2nd well, this step repeated from the 2nd well to the 3rd well till the 10th well. The prepared bacterial suspension (50 µL) was then added to each well followed by addition of 100 µL of TSB. Wells containing live bacterial cells without any tested compound and TSB without cells were included in each plate as positive and negative control, respectively. The plates were incubated at 37oC for 18 hrs, 40 µL resazurin (0.015 %) was then added to each well followed by incubation for further 2 hrs. The lowest concentration of each tested agent at which no bacterial growth was observed, with consequent unchanged blue resazurin color, was scored as the MIC.

Table S1. The minimum inhibitory concentrations (MICs in µg/mL) of the synthesized compounds initially screened against the indicated pathogens.

Code	P. aeruginosa ATCC 25668	P. aeruginosa ATCC 27583
**4a**	256 ± 0	1024 ± 0
**4a-NPs**	64 ± 0	1024 ± 0
**4b**	256 ± 0	1024 ± 0
**4b-NPs**	256 ± 0	128 ± 0

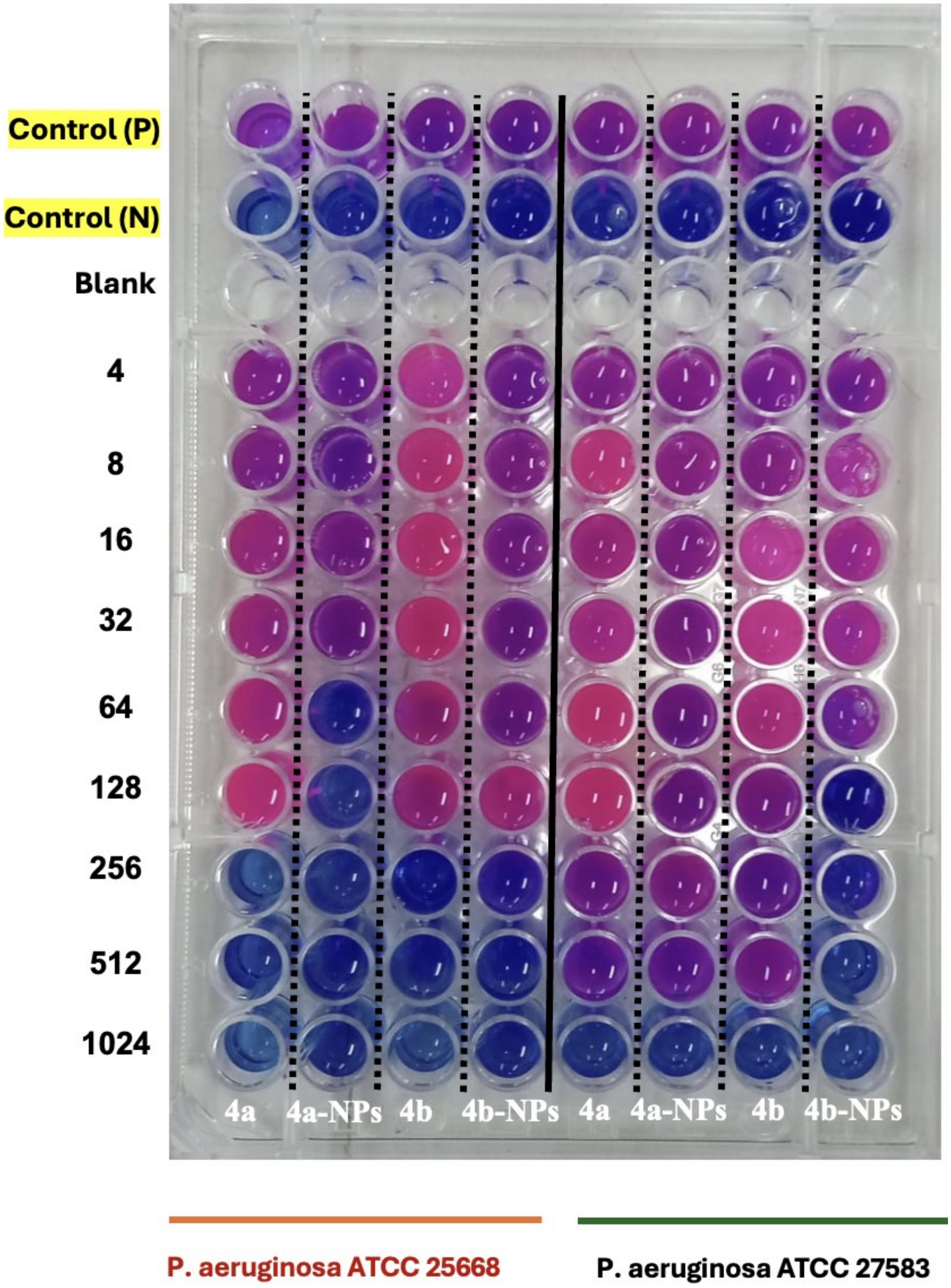

Figure S1: Antibacterial investigation of **4a**, **4a-NPs**, 4b, and **4b-NPs** against *P. aeruginosa* ATCC 25668 and ATCC 27583

Biofilm kinetics:

Experiment design

	1	2	3	4	5	6	7	8	9	10
conc ug/ml	0	1000	500	250	125	62.5	31.2	15.6	7.8	3.9
4a-NP	C	4a-NP	4a-NP	4a-NP	4a-NP	4a-NP	4a-NP	4a-NP	4a-NP	4a-NP
26-nano	C	4b-NP	4b-NP	4b-NP	4b-NP	4b-NP	4b-NP	4b-NP	4b-NP	4b-NP
mkh-27	C	4a	4a	4a	4a	4a	4a	4a	4a	4a
mklh-26	C	4b	4b	4b	4b	4b	4b	4b	4b	4b
Ciprofloxacin	C	Cip	Cip	Cip	Cip	Cip	Cip	Cip	Cip	Cip

XXT

	0	1000	500	250	125	62.5	31.2	15.6	7.8	3.9
27-nano	0.042	0.072	0.131	0.139	0.155	0.173	1.242	1.476	1.643	2.272
26-nano	0.061	0.096	0.165	0.174	0.186	0.964	1.351	1.559	1.873	2.249
mkh-27	0.037	0.103	0.155	1.119	1.421	1.552	1.589	2.207	2.229	2.376
mklh-26	0.033	0.157	0.939	1.187	1.354	1.529	1.641	1.859	1.972	2.283
Ciprofloxacin	0.055	0.089	0.143	0.169	0.176	0.942	1.219	1.476	1.675	2.175

crystal violet (CV)

	0	1000	500	250	125	62.5	31.2	15.6	7.8	3.9
27-nano	0.081	0.134	0.142	0.159	0.193	0.218	1.492	1.775	2.542	2.731
26-nano	0.079	0.161	0.183	0.221	0.239	1.425	1.777	1.937	2.284	2.594
mkh-27	0.066	0.147	0.222	1.239	1.272	1.341	1.875	2.042	2.296	2.661
mklh-26	0.064	0.158	0.963	1.376	1.752	1.991	2.3342	2.551	2.597	2.671
Ciprofloxacin	0.072	0.144	0.183	0.209	0.259	1.382	1.644	1.769	2.303	2.488

	4a-NP	4b-NP	4a	4b	Cip
XTTN = (XTT - ContXTT)/ContXTT	3.119	2.049	3.189	3.757	2.2
CVN = (CV - ContCV)/ContCV	1.691	2.025	2.363	1.468	2.59

XCR = XTTN/CVN

BSA = (XTTN/CVN) _ [(CVN + XTTN)/2]

BMV = XTTN _ CVN

	4a-NP	4b-NP	4a	4b	Cip
XCR=	1.844	1.012	1.349	2.558	0.847
BSA=	-0.561	-1.025	-1.427	-0.054	-1.551
BMV =	1.427	0.023	0.825	2.288	-0.397

The CV and XTT values were normalized according to Equation (1) and Equation (2) to determine the normalized values of CV and XTT, CVN and XTTN respectively,

CV(crystal violet)

XTT(XTT stain)

CVN = (CV - ContCV)/ContCV (1)

XTTN = (XTT - ContXTT)/ContXTT (2)

XCR = XTTN/CVN (3)

BSA = (XTTN/CVN) _ [(CVN + XTTN)/2] (4)

BMV = XTTN _ CVN (5)

The XCR parameter is a measure of the metabolic activity referring to the biofilm biomass and suffers of producing high values when the CVN is relatively low. It was, therefore, changed in the BSA by multiplying the XCR by the average of the XTTN and CVN, obtaining a descriptor of the XCR weighted on the average of the CV and XTT values. Finally, the BMV combines both the activity and biomass of the biofilm matrix in a single metric.

Cytotoxicity of nanoformulas 4a-NP and 4b-NP against the small molecules 4a – 4b and ciprofloxacin

	Blank	CC	Sample No. 4a-NP/WI38					Sample No. 4b-NP/WI38				
	1	2	3	4	5	6	7	8	9	10	11	12
A	B	C	1000ug	250ug	63ug	16ug	4ug	1000ug	250ug	63ug	16ug	4ug
B	B	C	1000ug	250ug	63ug	16ug	4ug	1000ug	250ug	63ug	16ug	4ug
C	B	C	1000ug	250ug	63ug	16ug	4ug	1000ug	250ug	63ug	16ug	4ug

ROBONIK P2000 Eia reader
Wavelength: 450 nm
Reference: 630 nm

	1	2	3	4	5	6	7	8	9	10	11	12
A	0.001	0.533	0.228	0.275	0.351	0.414	0.486	0.196	0.273	0.343	0.422	0.453
B	0.001	0.528	0.237	0.266	0.346	0.427	0.493	0.188	0.257	0.322	0.419	0.459
C	0.001	0.541	0.229	0.281	0.332	0.411	0.476	0.205	0.264	0.341	0.405	0.466
mean	4E-04	0.534	0.23133	0.274	0.343	0.4173	0.485	0.1963	0.26467	0.3353	0.415	0.459
%		`	43.3208	51.311	64.232	78.152	90.824	36.767	49.563	62.797	77.78	86.02

4a-NP/WI38

log conc.	% viability
3	43.32
2.4	51.31
1.8	64.23
1.2	78.15
0.6	90.82
IC50=	

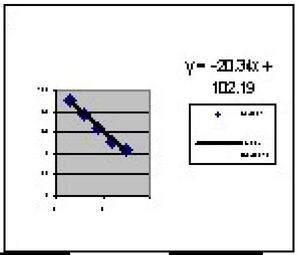

4b-NP/WI38

log conc.	% viability
3	36.7665
2.3979	49.563
1.7993	62.7965
1.2041	77.7778
0.6021	86.0175

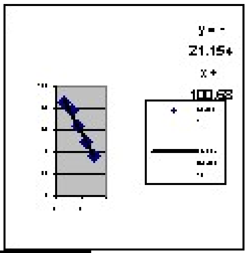

		[redacted]		[redacted]		[redacted]		[redacted]		[redacted]		

	Blank	CC	Sample No. 4a/WI38					Sample No. 4b/WI38				
	1	2	3	4	5	6	7	8	9	10	11	12

A	B	C	1000ug	250ug	63ug	16ug	4ug	1000ug	250ug	63ug	16ug	4ug
B	B	C	1000ug	250ug	63ug	16ug	4ug	1000ug	250ug	63ug	16ug	4ug
C	B	C	1000ug	250ug	63ug	16ug	4ug	1000ug	250ug	63ug	16ug	4ug

	**1**	**2**	**3**	**4**	**5**	**6**	**7**	**8**	**9**	**10**	**11**	**12**
A	0.001	0.553	0.225	0.288	0.331	0.377	0.424	0.186	0.251	0.328	0.376	0.424
B	0.001	0.537	0.209	0.275	0.316	0.379	0.427	0.179	0.238	0.313	0.381	0.431
C	0.001	0.541	0.227	0.276	0.321	0.368	0.431	0.193	0.244	0.322	0.363	0.425
mean	0.001	0.544	0.22033	0.2797	0.3227	0.3747	0.4273	0.186	0.24433	0.321	0.373	0.427
% viability			40.5273	51.441	59.35	68.915	78.602	34.212	44.9418	59.044	68.67	78.48

4a/WI38

log conc.	% viability
3	40.53
2.4	51.44
1.8	59.35
1.2	68.91
0.6	78.6
IC50=	

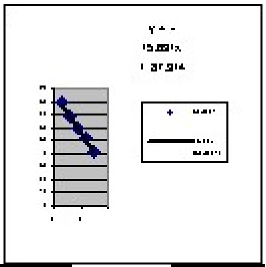

4b/WI38

log conc.	% viability
3	34.2121
2.3979	44.9418
1.7993	59.0435
1.2041	68.6695
0.6021	78.4795

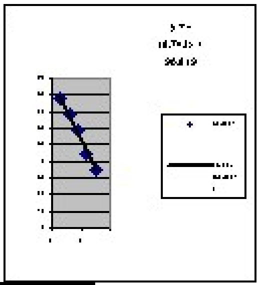

	**Blank**	**CC**	**Sample No.**			**CIP/WI38**		**Sample No.**				
	**1**	**2**	**3**	**4**	**5**	**6**	**7**	**8**	**9**	**10**	**11**	**12**
A	B	C	1000ug	250ug	63ug	16ug	4ug					
B	B	C	1000ug	250ug	63ug	16ug	4ug					
C	B	C	1000ug	250ug	63ug	16ug	4ug					

	**1**	**2**	**3**	**4**	**5**	**6**	**7**	**8**	**9**	**10**	**11**	**12**

A	0.001	0.616	0.228	0.292	0.366	0.413	0.441					
B	0.003	0.592	0.217	0.284	0.349	0.407	0.451					
C	0.001	0.603	0.231	0.303	0.361	0.392	0.433					
mean	0.002	0.604	0.2253 3	0.293	0.358 7	0.404	0.441 7	0	0	0	0	0
%viability			37.327 4	48.537	59.41 5	66.924	73.16 4	0	0	0	0	0

CIP/WI38

3	37.33
2.4	48.54
1.8	59.41
1.2	66.92
0.6	73.16

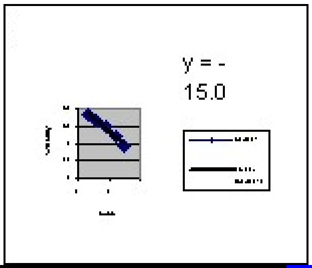

IC_{50}= [redacted]

Chemistry

Synthesis of Chlorothiolactone:[1]

Scheme 1

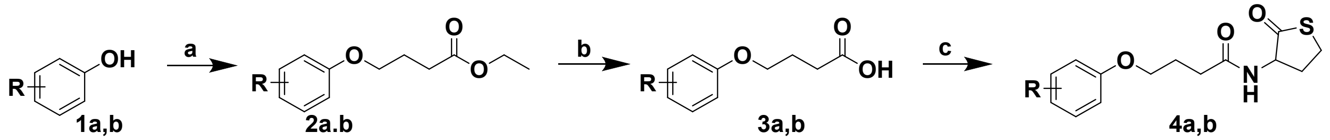

R: a) 3-Cl; b) 4-Cl

Reagents and Conditions: a) Ethyl 4-chlorobutanoate, K_2CO_3, Dry DMF, RT, 5 days; b) NaOH, THF:H_2O (3:1),RT, 12 h; c) 3-Aminodihydrothiophen-2(3*H*)-one hydrochloride,TEA, PyBOP, Dry DCM, RT, 12h.

The designated derivatives 4a-b were synthesized according to the following procedures

General Procedure (A) Alkylation step:

To a flame-dried flask was added, the appropriate phenol derivative (1.0 eq.), ethyl 4-chlorobutyrate (1.0 eq.), potassium carbonate (1.5 eq.), and dry DMF. The reaction was stirred at optimum temperature for 5 days or until completed (as indicated by TLC). After completion, cold water was added, and the aqueous layer was extracted three times with Et_2O (3×30mL). The combined organic layer was washed with NaOH (1 M) three times (to remove the residual phenolic starting material) and three times with brine followed by one time with brine. The solution was dried over anhydrous Na_2SO_4, filtered, and concentrated in vacuo to deliver the corresponding esters (**2a-b**) as yellowish oil which Was carried directly to the next step without further purification.

General Procedure (B) Ester hydrolysis step:

The obtained ester (**2a-b**) from the previous step (1.0 eq.) was dissolved in 15 mL THF: H_2O (3:1) followed by addition of NaOH (2M aqueous, 15 mL). The reaction stirred at room temperature for 12 h., or until completion (as detected by TLC). Following, solvent was concentrated under vacuum, and then the residue was extracted by DCM. The aqueous layer was collected, washed with another portion of DCM (10 mL) and acidified with HCl (1 M). The turbid solution was extracted with EtOAc (3×10 mL). The combined organic layers were washed with brine, dried over anhydrous Na_2SO_4, filtered, and evaporated to provide the corresponding acids (**3a-b**).

General Procedure (C) for Amide coupling step:

To a solution of **3a-b** (1.0 eq.) dry DCM (25 mL) triethylamine (1.0 eq.), 3-aminodihydrothiophen-2(3H)-one hydrochloride (1.0 eq.), Benzotriazole-1-yl-oxy-tris-pyrrolidino-phosphonium hexafluorophosphate (PyBOP) (1.5 eq.) was sequentially added. The reaction was stirred at room temperature for 12 h. After that, the reaction mixture was extracted by DCM and purified by column chromatography (methanol in DCM 0 to 10%) to afford the final products **4a-b** as off-white powder. The chemical structures and other data are shown below:

4-(3-Chlorophenoxy)-N-(2-oxotetrahydrothiophen-3-yl) butanamide (mCTL).

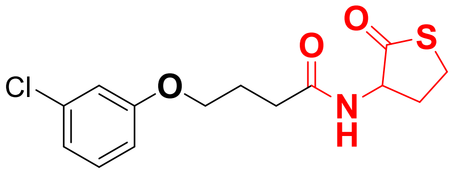

Following the general procedures A, B and C, and using 3-chlorophenol (0.5g, 3.9 mmol), mCTL was obtained as white solid powder (0.302g, 68 %) mp =57-59 °C; IR Cm-1: 3267 cm-1 (NH stretching), 2941 cm-1 (CH Aliphatic), 1743 cm-1 (C=O stretching), and 1457 cm-1 (CH_2 bending); ^{1}H NMR (500 MHz, DMSO-d^6) δ 8.21 (t, J = 6.9 Hz, 1H), 7.24 – 7.19 (m, 1H), 7.09 (q, J = 7.8, 5.4 Hz, 2H), 6.92 (t, J = 6.9 Hz, 1H), 4.59 (dq, J = 13.7, 6.8 Hz, 1H), 3.97 (q, J = 6.2 Hz, 2H), 3.26 (dd, J = 15.1, 8.8 Hz, 2H), 2.38 (dd, J = 12.5, 6.2 Hz, 1H), 2.26 (q, J = 7.0 Hz, 2H), 2.04 (dt, J = 12.4, 6.1 Hz, 1H), 1.90 (q, J = 6.7 Hz, 2H); MS (m/z) 314 (MH-,100%).

4-(4-Chlorophenoxy)-N-(2-oxotetrahydrothiophen-3-yl) butanamide (pCTL).

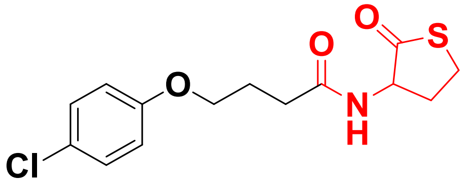

Following the general procedures A, B and C, and using 4-chlorophenol (0.5g,3.9 mmol), pCTL was obtained as white solid powder (0.271 g, 61 %) mp = 62-64 °C; IR Cm^{-1}: 3267 cm^{-1} (NH stretching), 2941 cm^{-1} (CH Aliphatic), 1743 cm^{-1} (C=O stretching), and 1457 cm^{-1} (CH_2 bending); ^{1}H NMR (500 MHz, DMSO-d^6) δ 8.22 (q, J = 9.3, 8.1 Hz, 1H), 7.30 (q, J = 8.3 Hz, 2H), 6.93 (tt, J = 7.8, 4.7 Hz, 2H), 4.60 (dp, J = 17.0, 8.2 Hz, 1H), 4.01 – 3.88 (m, 2H), 3.34 (dd, J = 24.6, 6.1

Hz, 2H), 2.38 (dd, J = 13.1, 6.8 Hz, 1H), 2.27 (p, J = 7.6 Hz, 2H), 2.05 (dt, J = 16.6, 8.3 Hz, 1H), 1.92 (dt, J = 14.7, 7.1 Hz, 2H); MS (*m/z*) 314 (MH^-,100%).

References:

(1) O'Loughlin, C. T.; Miller, L. C.; Siryaporn, A.; Drescher, K.; Semmelhack, M. F.; Bassler, B. L. A quorum-sensing inhibitor blocks Pseudomonas aeruginosa virulence and biofilm formation. *Proceedings of the National Academy of Sciences* **2013**, *110* (44), 17981-17986.

^{1}HNMR charts of compounds (4a-b)

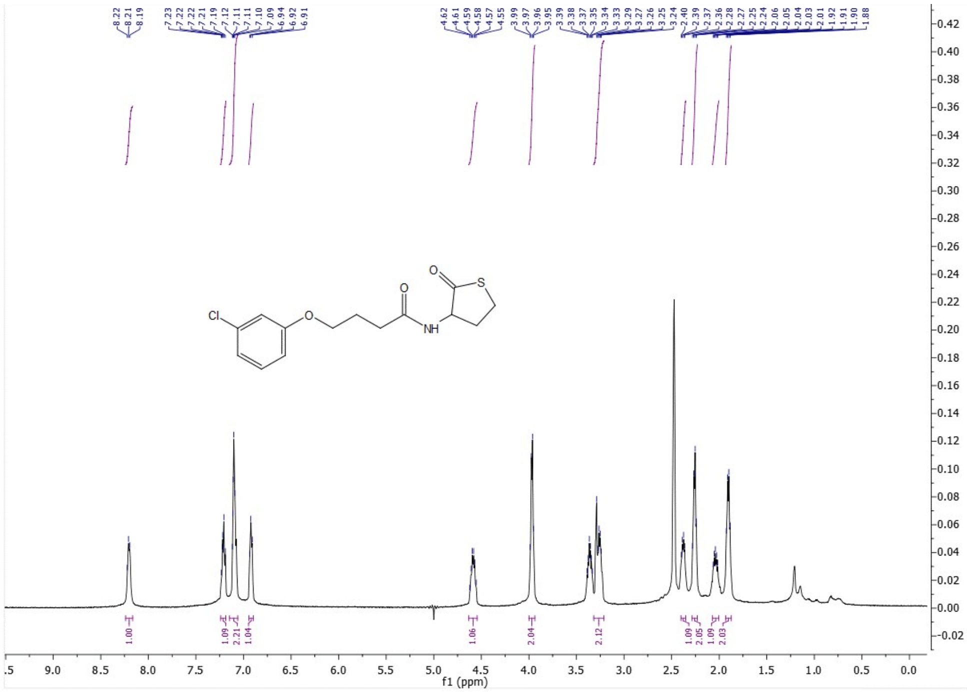

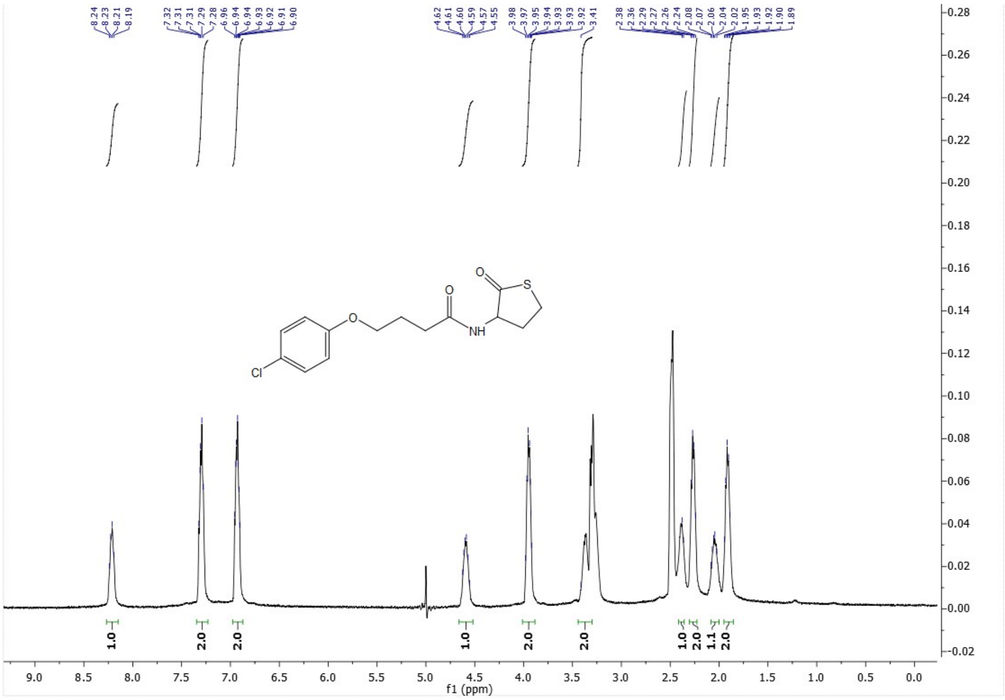

Supplement: RA-015-D5RA01831E-s001 [file RA-015-D5RA01831E-s001.pdf]
